# The Microenvironment’s Role in Mycosis Fungoides and Sézary Syndrome: From Progression to Therapeutic Implications

**DOI:** 10.3390/cells10102780

**Published:** 2021-10-17

**Authors:** Alessandro Pileri, Alba Guglielmo, Vieri Grandi, Silvia Alberti Violetti, Daniele Fanoni, Paolo Fava, Claudio Agostinelli, Emilio Berti, Pietro Quaglino, Nicola Pimpinelli

**Affiliations:** 1 Dermatology, IRCCS, S. Orsola-Malpighi Polyclinic, 40100 Bologna, Italy; albaguglielmo@gmail.com; 2 Department of Specialistic, Diagnostic and Experimental Medicine (DIMES), Alma Mater Studiorum, University of Bologna, 40100 Bologna, Italy; claudio.agostinelli@unibo.it; 3 Section of Dermatology, Department of Health Sciences, University of Florence, 20019 Florence, Italy; vieri.grandi@unifi.it (V.G.); nicola.pimpinelli@unifi.it (N.P.); 4 UOC Dermatologia, Fondazione Ca’ Granda Ospedale Maggiore Policlinico, 20122 Milan, Italy; silvia.viole@gmail.com (S.A.V.); daniele.fanoni@hotmail.it (D.F.); emilio.berti@unimi.it (E.B.); 5 Dermatologic Clinic, University of Turin Medical School, 10092 Turin, Italy; paolo_fava@yahoo.it (P.F.); pietro.quaglino@unito.it (P.Q.); 6 Hematopathology, IRCCS, S. Orsola-Malpighi Polyclinic, 40100 Bologna, Italy

**Keywords:** cutaneous, lymphomas, cutaneous T-cell lymphomas (CTCLs), Sezary syndrome

## Abstract

Background: Mycosis fungoides (MF) and Sezary Syndrome (SS) are the most common cutaneous T-cell lymphomas. It has been hypothesized that the interaction between the immune system, cutaneous cells, and neoplastic elements may play a role in MF/SS pathogenesis and progression. Methods: This paper aims to revise in a narrative way our current knowledge of the microenvironment’s role in MF/SS. Results and Conclusions: Literature data support a possible implication of microenvironment cells in MF/SS pathogenesis and progression, opening up new therapeutic avenues.

## 1. Introduction

**Figure 1:**
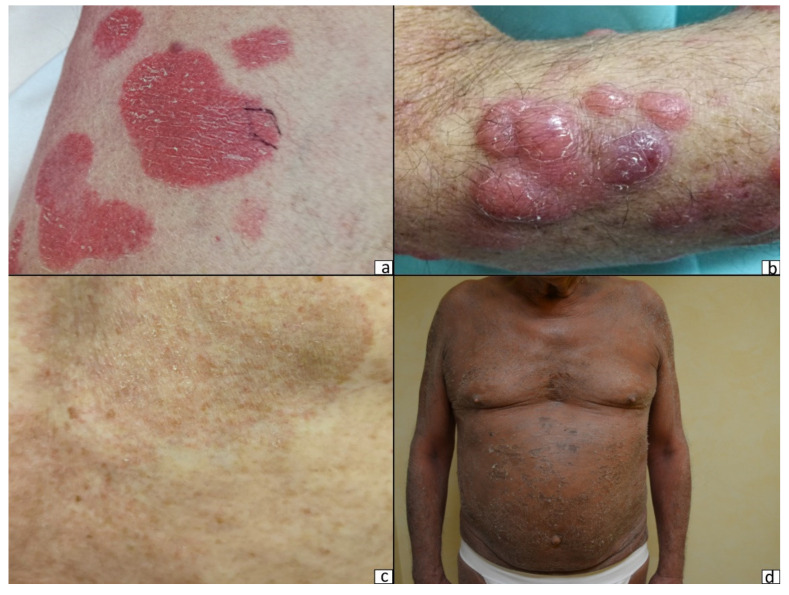
Different presentations of mycosis fungoides disease: (**a**) plaque stage, (**b**) tumor stage, (**c**) patch stage, and (**d**) erythroderma in a Sezary syndrome patient.

**Figure 2:**
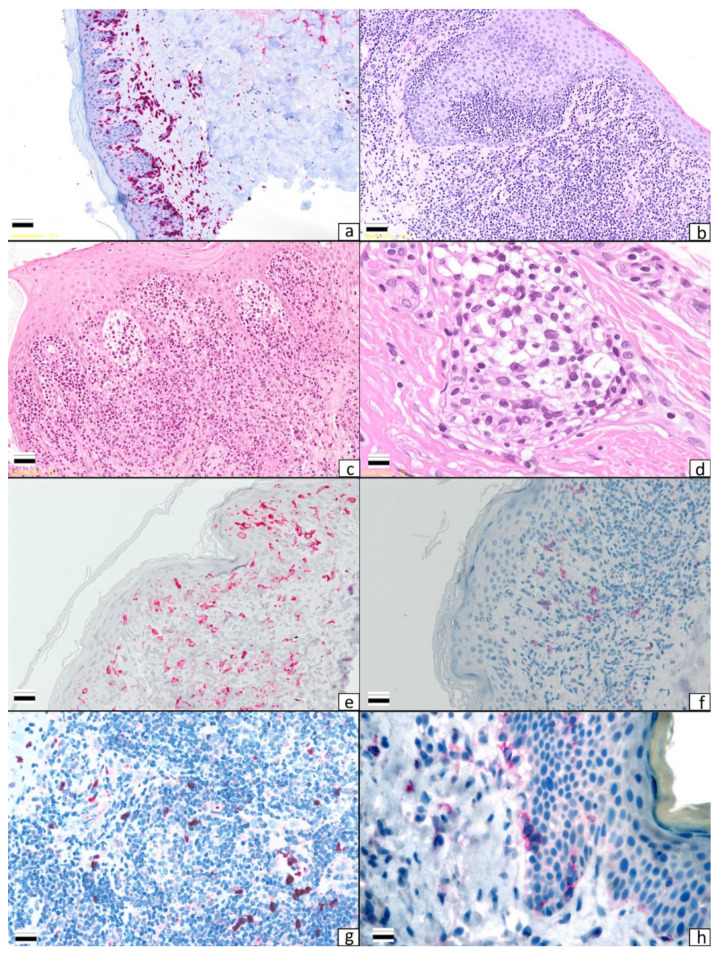
Different histological patterns in mycosis fungoides: (**a**) patch stage (×10), (**b**) plaque stage (×10), (**c**) tumor stage (×10), (**d**) Sezary cells within a blood vessel in a Sezary syndrome patient (×40), (**e**) a high number of Langerin-positive cells in an early stage of mycosis fungoides (×10), (**f**) CD303-positive cells in the plaque stage of mycosis fungoides (×10), (**g**) a high number of myeloid-derived suppressor cells (Arginase+) in the tumor stage of mycosis fungoides (×10), (**h**) a high number of VEGF-A-positive cells in a patch stage case (×10).

Mycosis fungoides (MF) and Sézary syndrome (SS) are the most common cutaneous T-cell lymphomas (CTCLs) [1]. Although MF and SS are closely related neoplasms, they are considered separate entities on the basis of differences in clinical behavior and the cells of origin. While Sézary syndrome (SS) is defined by the triad of erythroderma, generalized lymphadenopathy, and the presence of clonally related neoplastic T-cells with cerebriform nuclei (Sézary cells) in skin, lymph nodes, and peripheral blood, MF is characterized by a long-standing history of erythematous and scaly patch and plaque lesions, eventually evolving into erythroderma or tumor lesions (Figure 1 and Figure 2a–d). Such an evolution is fascinating from a clinical and therapeutic perspective owing to the different clinical outcomes (indolent in the early phases, aggressive in the advanced ones) and therapeutic approaches (skin-directed vs. systemic therapies). Although new treatment modalities have recently been proposed both for early [2] and advanced phases [3,4,5,6], the mechanisms involved in MF progression remain a matter of debate. It has been hypothesized that different players may be involved: aberrant molecular expression, genetic mutations, microRNA overexpression, changes in cytokine release, and different compositions of microenvironment cells [6].

The very first hypothesis that the immune system may reverse tumorigenesis and the spread of cancer cells to internal organs was proposed by Burnet et al. [7] in the 1950s. Since then, the immune system’s role has been investigated in many cancers. The tumor microenvironment can be defined as a complex system including cells and molecules that under certain conditions promote tumor growth and spread. Microenvironment cells can be defined as any type of cell that interacts with cancer cells to gain a specific phenotype and functions. Dunn et al. [8], in the 2000s, by proposing the so-called ‘immunoediting theory’, described three steps of interaction between neoplastic and microenvironment cells: elimination, equilibrium, and immune suppression. In the first step, the immune system reverses neoplasia in cells by inducing their apoptosis, leading to tumor destruction. In the case of failure, a sort of “equilibrium” between tumorigenic and anti-tumor actions can be observed. Such a balance between anti-tumor and tumorigenic actions will be lost later when the tumor cells acquire the ability to spread via the lymphatic and blood vessels. Under such circumstances, immunosuppression is predominant and induced by neoplastic cells by secreting immunosuppressive cytokines and recruiting immunosuppressive cells. An increased number of immature antigen-presenting cells within the microenvironment will be observed, leading to immune system anergy, the depletion of anti-tumor cells, and the accumulation of exhausted anti-tumor cells. Microenvironment changes in CTCLs have been the subject of several studies, and the aim of the present paper—which mainly focuses on MF—is to analyze the state of the art by reviewing the role of all microenvironment cells and analyzing all possible therapeutic approaches capable of reversing the microenvironment’s role from a tolerogenic to an anti-tumor one.

### 1.1. Dendritic Cells’ Role and Regulation in Anti-Tumor Immunity

First described by Langerhans in the late nineteenth century, the function of dendritic cells (DCs) has not yet been completely elucidated owing to the presence of different subsets featuring different functions. DCs act as the “sentinel” of the immune system and, as professional antigen-presenting cells (APCs), they activate naïve T-cells, orchestrating the innate and adaptive immune response [9]. However, DCs also induce tolerance by deleting self-reactive thymocytes, mediating the anergy of mature T-lymphocytes, and generating regulatory (Treg) cells [10]. The different actions exerted upon the immune system (activation/inhibition) are due to different DC subsets and different activation states. Immature DCs, by presenting antigens to T-cells in the absence of co-stimulatory signals (present on mature DCs), induce the development of Treg cells, eventually leading to tolerance [11]. Defective DC function has been related to many pathological conditions, such as autoimmune diseases, allergies, and cancers. In tumors, it has been hypothesized that the maturation state and location of DC infiltrates may be related to a different clinical outcome [10,12]. In humans, two main DC subsets can be observed: CD11c+, CD123_−_/low-myeloid DCs (mDCs) and CD11c-, CD123+ plasmacytoid DCs (pDCs). mDCs form the main group of professional APCs and can be observed in peripheral tissues, secondary lymphoid organs, and circulating blood. Two main populations of mDCs can commonly be observed in the skin: epidermal Langerhans cells (LCs) and dermal dendritic cells (DDCs). The former express the CD207/Langerin marker and the latter express the DC-DIGN/CD209 molecule [13]. pDCs are a unique cell population capable of producing large amounts of type I interferon (IFN) in the case of a viral infection. Type I IFN blocks viral replication, plays a pivotal role in linking the innate and adaptative immune system, and is fundamental to mDC activation. The pDC immunophenotype is characterized by the expression of CD123 and BDCA-2 (CD303) molecules [14].

### 1.2. Myeloid-Derived Suppressor Cells’ Role and Regulation in Anti-Tumor Immunity

Myeloid-derived suppressor cells (MDSCs) are a newly proposed cell population [15] whose nature and biological role have recently been clarified. They play a role as a regulator of the immune system response in many pathologic conditions. Two groups of MDSCs can be distinguished: granulocytic or polymorphonuclear MDSCs (PMN-MDSCs) and monocytic MDSCs (M-MDSCs). The former are phenotypically and morphologically similar to neutrophils, while the latter are more similar to monocytes [15]. Under chronic conditions and in cancers, there is a large accumulation of MDSCs featuring an immature morphology, weak phagocytic activity, and increased levels of arginase, nitric oxide (NO), and anti-inflammatory cytokines [16], leading to the inhibition of adaptative immunity and promoting tumor progression and spread. The most prominent factors implicated in MDSCs’ suppressive activity include high arginase and NO levels, upregulation of ROS, and the production of prostaglandin E2 (PGE2) [16]. MDSCs are thought to play an important role in cancer progression and high MDSC levels have been related to a worse clinical outcome in many cancers [17,18].

### 1.3. LCs, DDCs, and MDSCs in MF and SS

DCs’ role in MF has been the object of several studies posing the question as to whether DCs may play a crucial role in MF progression [10,19,20,21,22]. DCs are thought to attract tumor cells to the skin [23,24], eventually mediating the cross-presentation of tumor-related antigens within regional lymph nodes (Figure 2e). As a consequence, an expansion of effector T-cells will be observed. After expansion, anti-tumor lymphocytes will move to the skin as tumor-infiltrating lymphocytes (TILs) and will provide an anti-MF response after activation induced by costimulatory signals provided by infiltrating mature DCs. It has been postulated that the absence of or a reduction in mature DCs may lead to a dramatic defect in the above-described anti-tumor response, leading to tolerance and advantages in MF progression.

It has been hypothesized that LCs may play a role in MF and SS pathogenesis and progression. In 1976, Goos et al. [23] first proposed that LCs may play a role in MF pathogenesis owing to the detection of LCs in MF infiltrates, a finding later reported by two different groups [24,25]. In 2002, Luftl et al. [19], by comparing patch and plaque-stage MF to tumor-stage MF, found that equal numbers of immature and mature DCs were present in patch and plaque-stage MF. Most of the immature DCs were LCs observed within the epidermis, while equal numbers of mature and immature (CD1a+, CD1c+) DCs were interspersed between the lymphocytic infiltrates both in early and in advanced lesions. The authors’ conclusion was that DCs may play a double role, i.e., an anti-tumor role in early phases and a tumorigenic role in advanced ones. The induction of tolerance to tumor advantages may be due to the production of immunosuppressive cytokines, such as IL-10. Moving on, Schlapback et al. [10], by comparing MF tissue samples to healthy skin, found an increased level of DCs in MF patients. DCs interspersed within MF cells were mostly at an immature state (CD209/DC-SIGN1 DCs) and in close contact with tumor cells. The authors hypothesized that the accumulation of immature DCs may play a role in MF progression. Indeed, the accumulation of immature DCs may produce an immunosuppressive environment eventually facilitating tumor growth and spread. Moreover, by releasing chemotactic cytokines, MF cells may be able to recruit immature DCs from the blood, empowering the tumor immune escape response mechanisms. A further study [21] reached the same conclusions after analyzing the maturation state and distribution of DCs in 25 MF cases. A predominance of immature DCs over mature ones, as well as a positive correlation between the tumor infiltrate and DC numbers, was found. The authors [21] proposed that the inability of immature DCs to switch to a mature (and anti-tumor) state may be suppressed by MF cells. On the other hand, a defect in DCs’ activation may be the cause per se of an inefficient immune response and may explain the long course of the disease. The first finding of a positive correlation between the accumulation of immature DCs and MF progression was provided in 2014 [18]. The study described an increase in immature DC-SIGN+ DCs in tumor-stage MF compared with patch or plaque lesions. The authors’ conclusion was that immature DC-SIGN+ DCs induce immune suppression in the late stage of MF and may be a potential therapeutic target. The same conclusions have been further supported by other investigations [22] on the distribution of LCs, pDCs, and MDSCs in MF/SS tissue samples. A decrease in mature DCs (LCs in particular) was observed as well as an accumulation of immature DCs (pDCs) by comparing patch/plaque lesions to tumor lesions. Moreover, for the first time in the literature, in the same analysis an increase in MDSCs was detected. Taken together, these findings may reflect that the accumulation of immunosuppressive cells may be crucial in MF/SS progression (Figure 2f–g). This finding opened a debate on MDSCs’ role in MF/SS. Geskin et al. [26] observed a decrease in MDSC activity in IFN-responder patients, stressing the hypothesis of a possible role of MDSCs in MF/SS progression as well as MDSCs being a marker of response. The same conclusion was advanced by Argyropoulos et al. [27]. The authors observed that the presence of a high number of MDSCs featuring a granulocytic morphology could serve as a parameter for aggressive clinical behavior and, therefore, clinicians should provide aggressive treatment in such cases.

### 1.4. Regulatory B-Cells (Breg Cells)

Peripheral B-cells are a large category of cells featuring different phenotypic markers and functions [28]. B-cells are classically known to prompt an immune response and inflammation by promoting T-cell activation and proliferation as well as antibody production. In recent years, the presence of B-cells characterized by the ability to suppress the immune response has been postulated. The physiological role of those cells should be to avoid autoimmune diseases [29]. In analogy with regulatory T-cells (Tregs), regulatory B cells have been named Breg cells. Breg cells have the capacity to maintain immune tolerance via the release of immunosuppressive cytokines, such as IL-10, or by the expression of PD-L1 molecules [30,31,32]. Despite the efforts to analyze in detail the role and function of Bregs, the presence of Breg cells with different phenotypes remains a matter of debate. Different groups have described Breg cells with different phenotypes or roles [28,33,34,35,36]; however, it remains unclear whether Bregs should be regarded as a specific B-cell subset or whether, under unknown stimuli, B-cells acquire a suppressive function. However, a crucial indicator of Bregs seems to be the production of a large number of IL-10 cytokines.

### 1.5. Brigs in MF

It has been described that the presence of CD20+ tumor-infiltrating B-cells negatively modulates tumor growth in many different cancers and correlates to a better clinical outcome [37]. Other studies have shown that the presence of B-cells may be related to a worse clinical outcome and an empowerment of tumor angiogenesis [28]. Indeed, the presence of CD19+ infiltrating B-cells was related to a poor clinical outcome in ovarian cancer [38]. Other studies have shown that the presence of CD20+ B-cells and CD138+ plasma cells also had the same negative prognostic impact on ovarian neoplasms [39]. Such contrasting findings on B-cells’ prognostic impact in cancer remain a matter of debate. In MF, little is known and only a few reports are available. Akatsuka et al. [40] observed a decrease in CD19+ CD24hiCD27+ B-cells, CD19+ CD38hi B-cells, and IL-10-producing B-cells related to advanced MF phases. Their conclusion was that the decrease in IL-10-producing Bregs may play an important role in MF progression. Some case reports of MF patients with the CD20+ phenotype with a good response to rituximab (an anti-CD20 monoclonal antibody) are available in the literature [41,42], suggesting that Bregs may play a role in MF progression and encouraging further studies to shed light on their role.

### 1.6. Regulatory T-Cells (Treg Cells)

Regulatory T-cells (Tregs) are a T-cell population capable of negatively modulating the activity of other lymphoid cells. Tregs represent approximately 5%–10% of peripheral T-cells and express a CD4+ CD25+ phenotype, while their physiological role is to avoid autoimmune diseases and induce tolerance to self-antigens [43]. Tregs express the transcriptional repressor Forkhead box P3 (FOXP3), which is considered to be a specific marker of this T-cell subpopulation. However, Tregs play an important role in immune evasion by neoplastic cells, empowering tumor immune escape response mechanisms. In several types of cancer, an increase in Tregs is related to a worse clinical outcome, while in many hematologic diseases high numbers of FOXP3+ cells are related to a good prognosis with improved survival rates [44].

### 1.7. Tregs in MF and SS

Contrasting results on Tregs’ role in MF are present in the literature. At first, Berger et al. [45] proposed that MF cells may have a Treg phenotype, and so MF may be regarded as Treg neoplasia. Such a theory was further supported by others who observed an expression of FOXP3 in five MF cases with a large cell transformation [46]. However, the availability of a more specific FOXP3 antibody was crucial for refuting the initial speculation. Indeed, several groups demonstrated that FOXP3 expression is rare in neoplastic cells, while FOXP3+ cells are mainly non-neoplastic cells [47,48,49,50,51,52,53,54]. Most of the studies provided evidence of the presence of a high number of Treg cells in the early patch/plaque stage, while in advanced MF phases the number of FOXP3+ cells was lower [47,48,49,50,51,52,53,54,55]. In addition, Gjerdum et al. [49] related an increased Treg number to a better clinical outcome. Another study found low numbers of FOXP3+ cells both in tissue samples and in blood samples of Sézary syndrome (SS) patients [47], supporting the evidence provided by Gjerdum et al. [49]. The same findings at the molecular level were made by Johnson et al. [56] by observing a decreased expression of FOXP3 mRNA in skin samples in advanced MF stages. However, some studies provided criticism of the prognostic role of Treg cells in MF. Indeed, Fried et al. [54] found a non-stage-dependent expression of FOXP3 in 14 patients with sequential biopsies, evidence not confirmed by Zhang et al. [57]. However, the theory that Treg cells may not only suppress the anti-tumor response, as observed in solid malignancies, but may also negatively modulate MF cell growth contrasts with the finding that some treatments, such as mogamulizumab or lenalidomide, may decrease the numbers of FOXP3+ cells in treated patients [58,59,60]. Geskin et al. [26] added to our current knowledge the finding that Treg counts in SS patients may be underestimated. Indeed, by analyzing CD4+ CD25+ T-cells isolated from blood samples, the authors found a high level in Treg cells. The authors speculated whether in previous reports Treg counts may have been miscalculated due to the expansion of the malignant clone. Moreover, in their analysis the American group [26] found a connection between the Treg cell number and MDSC activity, suggesting crosstalk between the two populations. Taking all the studies together, it is clear that Tregs’ role in MF as well as in SS is yet to be fully elucidated.

### 1.8. Macrophages

Macrophages play important roles in inflammation (cytokine release, phagocytosis) and tissue repair (stem cell proliferation, angiogenesis, fibrosis). So-called “macrophage polarization” is a concept that explains how macrophages can be directed towards inflammatory or reparative functions by different stimuli from their microenvironments. Macrophages are a component of the innate immunity and are one of the major players in the leukocyte infiltrate [61]. Macrophages involved in tumor development are called tumor-associated macrophages (TAMs) and play a critical role in the biology of various types of cancers. Based on the response to various stimuli from tumor cells, macrophages are polarized into two main categories: M1 (classically activated) and M2 (alternately activated) [62]. M1 macrophages are induced by interferon (IFN)γ and have anti-tumor activity, producing inflammatory cytokines such as interleukin (IL)-1β, tumor necrosis factor (TNF)-α, IL-6, and IL-23. M2 macrophages are induced by IL4 and promote a Th2 immune response and tumor growth and progression [62,63]. These two phenotypes are not stable and, considering the plasticity of macrophages, several in vivo studies have demonstrated that the polarization can change over time [64]. A high number of TAMs in the tumor microenvironment correlates to poor survival in patients with several cancer types, including hematological malignancies, such as follicular lymphoma, angioimmunoblastic T-cell lymphoma, Hodgkin’s lymphoma, and diffuse large B-cell lymphoma [65]. In particular, M2 TAMs are involved in immune suppression, tumor migration, invasion, and angiogenesis by releasing vascular endothelial growth factor (VEGF), matrix metalloproteinase (MMP)-7, MMP-9, IL-12, high levels of IL-10, TGF-β, hepatocyte growth factor and basic fibroblast growth factor, adrenomedullin, urokinase-type plasminogen activator, thymidine phosphorylase, prostaglandin E2, and semaphoring 4D [66]. Moreover, the subpopulation M2a, by IL4 and IL13 induction, promotes the expression of chemokine ligand (CCL) 24, CCL17, and CCL22, favoring the recruitment of eosinophils, basophils, and Th2 cells. The M2b subpopulation secretes CCL1, a chemokine that, with its receptor CCR1, promotes the infiltration of eosinophils, Th2 cells, and T regulatory (Treg) cells. Finally, the M2c subpopulation, by IL10 stimulation, expresses chemokine ligand (CXCL) 13, CCL16, and CCL18, which with their receptors CXCR5, CCR1, and CCR8 induce the accumulation of eosinophils and naïve T-cells with T-cell anergy [64].

### 1.9. Tumor-Associated Macrophages (TAMs) in MF and SS

The role of TAMs in the cutaneous T-cell lymphoma (CTCL) microenvironment has been also reported, showing that M2 TAMs are involved in the development and progression of CTCL. In their xenograft human CTCL cell model, Wu et al. [67] demonstrated an important role of macrophages in tumor development and lymph angiogenesis. The authors injected MBL2 T lymphoma cells into the ears of mice and induced lymphoma development by application of di-nitro-fluorobenzene. After checking for the presence of numerous M2 macrophages in the lesions, they used a clodronate-encapsulated liposome to selectively remove the macrophages and showed a strong reduction in skin tumors. Moreover, in the group of clodronate-treated mice, STAT3 was found to be down-modulated by podoplanin and CD31, which stained lymphatic and vascular vessels. By the expression of CD163, a marker of M2 macrophages, Sugaya et al. [65] demonstrated that M2 cells were significantly more numerous in CTCL lesional skin compared with normal skin, and their number increased as more tumor cells infiltrated. These data also correlated with a worse prognosis. Similarly, the serum of CTCL patients showed significantly higher levels of CD163 than those of normal controls, correlating also with serum IL-2R levels. M2 TAMs have also been correlated to lymph-node staging [68]. Indirectly, the role of macrophages in CTCL development was also demonstrated by the evaluation of CCL18, a chemokine involved in inflammatory skin reactions, by the recruitment of Th2 cells with CCL17 and CCL26. Previous in vitro studies showed the contradictory effects of CCL18 on CTCL cell lines [69]. Instead, Miyagaki et al. [70] showed high in vivo serum and skin levels of CCL18 in CTCL compared with controls and these data significantly correlated to modified severity-weighted assessment scores, serum sIL-2R, and a poor prognosis. The involvement of macrophages in CTCL has also been studied in early stages. Furudate S et al. [71] evaluated the activity of macrophages in each stage of MF, from the early stage to the tumor stage, and the role of periostin, an extracellular matrix protein that is known to be involved in the recruitment of Th2 cells and the polarization of M2 macrophages in the tumor microenvironment [72,73]. The authors found that in plaque-stage MF, periostin-stimulated macrophages are the dominant factor in the formation of the tumor mass and, after the plaque stage, M2-like macrophages are dominant in maintaining an immunosuppressive tumor microenvironment [71].

### 1.10. Keratinocytes

Keratinocytes represent 90% of the cell types present in the epidermis. Their function is to provide a barrier against external agents. However, keratinocytes play a role in immune system activation [74]. Epidermal keratinocytes express several tool-like receptors (TLRs). TLR expression by keratinocytes may be crucial for promoting skin immune responses, as the activation of these receptors on human keratinocytes leads to a predominant Th1-type immune response and to the production of type I interferon (IFN). Moreover, by expressing MHC class II molecules, keratinocytes act as non-professional APCs and it has been supposed that keratinocytes may display features of APCs with the potential for both antigen-specific tolerization and activation.

### 1.11. Keratinocytes in MF and SS

It has been hypothesized that interaction and cross-signaling between keratinocytes, stromal cells, and malignant T-cells may lead to MF and SS progression [74]. Different studies have highlighted the existence of a complex loop of continuous signaling between keratinocytes, fibroblasts, and malignant T-cells that eventually leads to the permanent activation of STAT proteins and induces the expression of tumorigenic (Th2) molecules [74]. Takahashi et al. demonstrated that a feedback loop between keratinocytes, stromal cells, and malignant T-cells leads to the activation of the STAT gene, resulting in a Th2 polarization of the inflammatory milieu. The same change within the microenvironment’s composition has the consequence of reinforcing STAT pathway expression [75]. STAT overexpression can also be induced by periostin, a molecule expressed by fibroblast elements. Different groups have observed high levels of periostin and thymic stromal lymphopoietin (TSLP) in both the serum and the lesional skin of CTCL patients [70,75,76]. Theoretically, high Th2 cytokine levels can induce periostin expression in dermal fibroblasts, stimulating epidermal keratinocytes to produce TSLP [75]. TSLP can induce STAT5 activation in malignant T-cells, promoting their survival and proliferation via IL-4 and IL-13 overexpression [75]. Furthermore, STAT5 overexpression downregulates STAT4 and SATB1 expression through the induction of microRNA-155 (miR-155) [77,78,79], empowering tumor immune response escape mechanisms and decreasing the secretion of anti-tumor molecules, such as IFNγ. Moreover, high periostin, IL-4, and IL-13 levels may stimulate the production of other immunosuppressive molecules, such as IL-25 [80,81]. High IL-25 levels have been observed in advanced MF and SS. Theoretically, high IL-25 levels may empower the expression of immunosuppressive cytokines, such as IL-13, via activation of the STAT6 pathway [81]. In conclusion, all of the above-mentioned findings illustrate that, after an initial increase in Th2 cytokines induced by neoplastic elements, the crosstalk between keratinocytes, fibroblasts, stromal cells, and malignant T-cells can start a complex loop of continuous signals that sustain and empower tumorigenic and depauperate anti-tumor action in CTCL [82].

### 1.12. Endothelial Cells

Angiogenesis is the process of formation of new blood vessels from existing ones and the same process involving lymphatic vessels is called lymph angiogenesis. Development of new vessels is complex and consists of different steps: migration, proliferation, and differentiation of endothelial cells, extracellular matrix degradation, and formation/stabilization of new vessels. All these steps are regulated by growth factors, cytokines, and other proteins. Neo-angiogenesis and lymph angiogenesis can be analyzed in different ways: by investigating micro-vessel density (characterized by MMP-2, MMP-9, and CD34 expression) or by analyzing vascular endothelial growth factor (VEGF) expression (VEGF-A for angiogenesis and VEGF-C for lymph angiogenesis).

### 1.13. Endothelial Cells in MF and SS

It has been speculated whether an increase in both angiogenesis and lymph angiogenesis may be related to MF progression. Angiogenesis in MF has been extensively investigated over the last three decades (Figure 2h). In 1997, Vacca et al. [83] found that the micro-vessel density (MVD) was higher in MF than in healthy controls by analyzing MMP-2 and MMP-9 mRNA levels. Moreover, the highest MVD was observed in tumor lesions. The same findings were later reported by Rasheed et al. [84] and Mazur et al. [85]. The latter [85] found that the mean number of CD34+ endothelial cells was significantly higher in MF than in normal skin samples. The increase in microvascular density levels were corroborated by further studies [86] demonstrating an overexpression in VEGF-A levels both at the protein and molecular level. The role of lymph angiogenetic markers has been analyzed by different authors. Karpova et al. [87] investigated lymph angiogenic marker expression (CD31, podoplanin, LYVE-1, VEGF-C, and VEGFR-3) in MF and SS cases. The authors found high levels of the selected markers, speculating a possible role in MF and SS pathogenesis. Jankowska-Konsur et al. [88,89] reported that an increase in density in lymphatic vessels was related to an increased possibility of lymph node metastases. The authors analyzed podoplanin and VEGF-C expression in MF and SS. Podoplanin, a glycoprotein expressed in the lymphatic endothelium, is related to the expression of vascular endothelial growth factor C (VEGF-C), a molecule that is considered to be a key stimulator of lymph angiogenesis [89,90]. Due to the presence of high podoplanin and VEGF-C levels in advanced MF and SS lesions, the authors proposed those markers as being related to aggressive clinical behavior. The same conclusion has recently been reached by other investigations [91] focused on lymphotoxin α (LTα)’s role in the formation of lymphatic vasculature and secondary lymphoid structures in CTCL. LT-α is involved in the regulation of cell survival, proliferation, differentiation, and apoptosis, can exert an anti-tumor or a tumorigenic function, and, as a pro-cancerogenic molecule, can play a role in angiogenesis. It has been observed that the expression of in situ LTα in CTCL cells is driven by an aberrantly activated JAK3/STAT5 pathway. LTα may act as an autocrine factor by stimulating the expression of IL-6 in malignant cells. Theoretically, LTα, IL-6, and VEGF may promote angiogenesis, inducing an increase in endothelial cells and, thus, promoting tumor growth and spread [92].

### 1.14. Tumor-Infiltrating Lymphocytes (TILs)

Tumor-infiltrating lymphocytes (TILs) represent a heterogeneous population of T-, NK, and B-cells activated against tumor cells. Infiltration of immune cells, particularly infiltration of anti-tumor type 1 lymphocytes, is associated with an improved prognosis in many different tumor types. However, due to immunosuppressive factors within the tumor microenvironment (TME), their tumor-killing ability is inhibited. Therefore, TILs exert a major role in the response of the immune system to tumor cells.

### 1.15. Tumor-Infiltrating Lymphocytes in MF and SS

An unusual and, therefore, fascinating feature of MF is that TILs have to control a malignant population from within their own lineage [93]. Unfortunately, studying the TILs in the MF microenvironment is technically challenging. The main problem, apart from the rarity of the condition, is that no single positive surface biomarker is able to accurately separate MF cells from the reactive, benign CD4+ T lymphocytes. Hence, all the methods used so far to distinguish MF cells from reactive CD4+ cells, except possibly for single-cell DNA analysis paired with a TCR repertoire, are hampered by a selection bias, sometimes potentially collecting only some of cells from a more heterogeneous tumor population, or by mislabeling a some of the reactive cells as cancerous. Taking into account these important technical limitations, multiple studies have highlighted the progressive shift from a Th1-enriched TME, as observed in early stage MF skin lesions, towards a Th2-oriented TME in the advanced stages, with the loss of Th1 markers and activated CD8+ cells and increased expression of Th2 markers, such as GATA3, IL-4, IL-5, and IL-13 [56,77,82,94,95,96,97,98]. Hence, these discoveries drove great expectations for IL-4 inhibitors as promising therapies in CTCL. Unfortunately, the use of the IL-4 inhibitor dupilumab (Dupixent) in patients with MF or SS may lead to rapid disease progression [99,100]. Hence, the “Th1 to Th2 disease progression” theory is likely an oversimplification. More data are needed, especially from early stage MF samples, to better understand, and potentially exploit, cytokine modulation in CTCL. More recent studies have proposed that the leading modification occurring in TILs of the MF TME is immune exhaustion. T-cell immune exhaustion is a dysfunctional status of T lymphocytes defined, classically, by the coexistence of a reduced cytotoxic activity, a decreased ability to secrete cytokines, increased expression of inhibitory receptors, reduced proliferation, and a reduced survival rate [101]. More recently, it has been proven that the exhausted phenotype is best described by its unique transcriptomic signature. In particular, the key role of thymocyte-selection-associated high-motility group box protein (TOX) in regulating the exhausted phenotype of CD8+ T-cells [102], and likely of CD4+ T-cells, has been postulated. TOX is a nuclear binding protein that plays a fundamental role in in the maturation of T-cells and NK cells but is also critical to the differentiation of tumor-specific T-cells [103]. Chronic TCR signaling is the main driver of immune exhaustion. During this process, TOX has been proven to induce immune exhaustion by increasing the expression of several exhaustion-related genes and suppression of effector-related ones, and it has been demonstrated that exhausted T-cells do not form in its absence [104]. Multiple studies focused on the genomic and transcriptomic profile of CTCL by WES, and WGS and bulk RNA-Seq have highlighted recursive perturbations on multiple points in the TCR-signaling machinery [105,106,107,108,109,110,111]. Most of these alterations converge into an abnormal chronic activation status. In particular, there is a growing body of literature showing the increased expression of TOX in CTCL both by tumor cells and by CD4+ and CD8+ reactive T-cells in skin and blood samples [112,113,114]. Perhaps not surprisingly, TOX is a good biomarker on immunohistochemistry to differentiate CTCL from benign inflammatory dermatoses. [107,115]. It has been identified that CD4 and CD8+ TILs and tumor cells obtained from lesional skin samples in MF cases show increased co-expression of the typical exhausted T cell surface markers PD-1, TIGIT, and TIM-3 [93]. This increase was not shared with paired T-cells from PBMCs, suggesting a local modulation rather than a generalized immune exhaustion. The degree of expression of these markers was broadly uniform among patients and among stages, suggesting that this immune exhaustion may occur as an early event in MF biology and as a commonly affected pathway.

### 1.16. NK Cells

NK cells work as the effector branch of the innate immune system and undergo activation in response to a reduction or complete abrogation of the self-human leukocyte antigen (HLA-I) alleles on tumor cells, with the release of granzyme and perforin alongside the production of IFN-gamma and TNF alpha [116]. NK cells are the best-studied elements among the innate immune system, and their anti-tumor role has been extensively studied. They are the most abundant innate immune cells, and their numbers correlate directly with a better prognosis and a reduced risk of metastasis [117,118].

### 1.17. NK Cells in MF and SS

NK cells may strongly express PD-1 (in a KIR+ NKG2A-CD57+ subpopulation) in PBMCs in healthy donors, and their count is stable over time, regardless of the donor’s age [119]. Their increase in the TME (but not in the paired PBMCs) has been proven to be associated with a worse prognosis in several solid and hematologic tumors, but not yet in MF or SS. [120,121,122] These NK cells have shown increased anti-tumor activity against PD-1/PD-L2+ tumor cells and may be relevant to the treatment of tumors showing a T-cell-resistant phenotype. Hence, one approach to improve the responses would be to combine an anti PD-1 or PD-1L agent with a checkpoint inhibitor related to NK cells. NK cells also express Killer Immunoglobulin Receptors (KIRs), which can be functionally divided into inhibitory (iKIRs) and activating (aKIRs). In particular, NK cells recognize and kill cells lacking or poorly expressing the ligand of their iKIR (“missing self-hypothesis”). Hence, these cells are extremely important in recognizing tumors downregulating HLA-I molecules. On the other hand, if a tumor does not downregulate its HLA-I, iKIRs aid the immune evasion; thus, blocking the iKIR–HLA interaction can boost the immune response against HLA-I+ tumors. [123] NKG2A is an inhibitory receptor co-expressed with CD94 in about 50% of NK cells in the peripheral blood [124]. CD94 and NGK2A work by recognizing HLA-E, which is ubiquitously expressed on normal human tissue, and its interaction with CD94/NGK2A determines a strong inhibition of the activating receptor NKG2C [125]. Hence, this is another “self-signaling” mechanism inducing self-tolerance and preventing NK-mediated autoimmunity. Unfortunately, tumor cells might upregulate the expression of HLA-E, effectively reducing the anti-tumoral NK and other cytotoxic lymphocyte-mediated responses, leading to a worse prognosis [126,127,128,129,130,131]. Hence, targeting the NGK2A axis seems to be a clever approach to reversing cytotoxic inhibition in several malignancies. However, there is a lack of data on NK cells’ role in the TME and progression in MF. Sako et al. [132] observed an increase in expression of the NK receptor KIR3DL2 (CD158k) in MF, proposing the hypothesis that MF cells may originate from a subset of NK cells expressing CD160 and KIR3DL2.

### 1.18. Eosinophils

Eosinophils are innate immune cells involved in the protective immune response of the host against helminths and viral and microbial pathogens. Human eosinophils derive from CD34+ CD117+ pluripotent hematopoietic stem cells in the bone marrow, where they complete their maturation and subsequently enter the bloodstream [133]. Phenotypically, eosinophils are characterized as CD11b+/Gr-1lo/F4-80+ cells. These markers are also found on macrophages, but eosinophils can be distinguished due to their high granularity, lack of expression of MHC-II, and expression of the sialic-acid-binding lectin Siglec-F [134]. Eosinophils are recruited from the blood into the sites of inflammation where, upon activation, they can release an array of inflammatory mediators, such as cationic proteins (major basic protein (MBP) and eosinophil cationic protein (ECP)), eosinophil peroxide (EPX), and eosinophil-derived neurotoxins (EDNs), that are unique to eosinophils and are important in the defense against parasitic infections [135]. Worth noting is that IL-5, IL-3, and GM-CSF are crucial for supporting the maturation of human eosinophils in the bone marrow [136] and mediate their survival by NF-κB-induced Bcl-xL, which inhibits apoptosis.

### 1.19. Eosinophils’ Role in MF and SS

Evidence indicates the presence of eosinophils in the TME of several human hematological and solid tumors, even if the mechanisms responsible for the infiltration of eosinophils into the tumors are not completely known [137,138,139]. However, some data show that the high-mobility group box 1 protein (HMGB1), IL-1α, and IL-33 potentially trigger eosinophil recruitment [140]. Moreover, macrophages and MCs can recruit eosinophils via the production of VEGFs [141,142] and/or the release of histamine and prostaglandin D2 (PGD2) through the activation of the chemoattractant-homologous receptors expressed on Th2 cells (CRTH2) [143] and H4 receptors [144], respectively. In the TME, eosinophils influence other leukocytes, such as T-cells, NK cells, DCs, and macrophages. In particular, they are able to recruit and activate T-cells through CXCL9, CXCL10, and CCL5, attract NK cells by IL-6, IL-12, and CXCL10 production, and induce M1 polarization [145]. Therefore, the presence of eosinophils in the tumor or in the bloodstream is a favorable prognostic factor for many cancers, although evidence of a pro-tumorigenic role for eosinophils has been reported [146]. Recent findings revealed that eosinophils display regulatory functions towards other immune cell subsets in the TME or direct cytotoxic functions against tumor cells, leading to either anti-tumor or pro-tumor effects. This paradoxical role of eosinophils was suggested to be dependent on the different factors in the TME [145]. Usually, in CTCLs eosinophils are rare within the infiltrate. Some authors have described a significantly higher number of eosinophils in the advanced stages than in the early stages, while other studies did not find correlations between the number of these cells and the stage of the disease. In 2016, Iliadis et al. observed a virtual absence of eosinophils in the early MF stage. In the study, there was no statistically significant correlation between the number of eosinophils and the stage of the disease, nor between the number of cells and the treatment response [147]. Other authors did not find significant correlations between the number of infiltrating eosinophils and the disease stage in MF [148]. However, many studies proposed an active role for eosinophils within the infiltrate in CTCL [149,150]. Ionescu et al. suggested that both the density and activation of tissue eosinophils were significantly related to disease progression in 26 primary CTCLs (including MF cases) with blood eosinophilia [151]. Theoretically, an accumulation of eosinophils in the TME may be related to disease progression, indicating a pro-tumor role of eosinophils in CTCLs or, at least, no anti-tumor actions. A suggested mechanism in which STAT3 activation in neoplastic T-cells leads to eosinophil accumulation in the TME through IL-5 production by malignant T-cells was hypothesized by Fredholm et al. The authors speculated as to whether malignant T-cells may “trap” inactivated eosinophils by a high secretion of cyclooxygenase 2 and prostaglandin E2 (PGE2) [152]. Finally, a massive presence of eosinophils in the tumor infiltrate was observed in erythrodermic mycosis fungoides (E-MF) [153] and in follicular mycosis fungoides (F-MF) [154,155].

### 1.20. Fibroblasts

Fibroblasts (FBs) are cells that synthesize and integrate structural proteins, such as collagen and elastin, into the extracellular matrix (ECM) of most mesenchymal tissues. Furthermore, FBs play an essential role in maintaining the structural integrity of most tissues. As a consequence, it is not surprising that cancer-associated fibroblasts (CAFs) are the most abundant type of cells within the tumor microenvironment (TME). They are activated fibroblasts that share several similarities with fibroblasts found in fibrotic tissues or during the healing phase of a wound [156]. Their presence in a tumor is associated with a poor prognosis in several types of cancer [157,158,159]. The recruitment of activated fibroblasts in many cancers is dependent on transforming growth factor beta (TGFβ) [160,161]. Local CAF proliferation and invasion is stimulated by TGFβ secretion by TME cells. Moreover, in the TME, CAF-derived TGFβ plays a key paracrine role in controlling epithelial carcinogenesis. More specifically, TGFβ secreted by CAFs promotes the epithelial-to-mesenchymal transition (EMT) process by weakening intercellular epithelial adhesion [162]. Furthermore, CAF-derived TGFβ stimulates the EMT in the adjacent cancer cells in various types of cancer [163,164]. Activation of fibroblasts could reflect a host defense mechanism to restrain cancer progression and potentially eliminate cancer [165,166,167,168]. Nevertheless, in solid tumors, the ability of CAFs to influence tumor growth was partly dependent on their ability to induce angiogenesis by CXCL12 and to recruit bone-marrow-derived endothelial cells [169]. Theoretically, in the early stages of neoplasia, inflammatory cues, emerging from pathological tissue remodeling, may initiate pro-inflammatory and tumor-promoting functions in fibroblasts. IL 1β secretion by immune cells in early lesions emerges as a potential initiator of nuclear factor κB (NF κB) signaling in fibroblasts, instructing them to produce a pro-tumorigenic secretome [170].

### 1.21. Fibroblasts’ Role in MF and SS

In CTCLs, the presence and role of CAFs remain unknown. In a recent study, Aronovich et al. sought to characterize CAFs in MF using primary fibroblast cultures from punch biopsies of patients with early stage MF. They found increased levels of CAF-associated genes and proteins, particularly CXCL12, collagen XI, and MMP2. MyLa cells cocultured with MF-derived fibroblasts reduced their sensitivity to doxorubicin and enhanced their migration. The authors speculated whether CAFs may protect MF cells from doxorubicin-induced cell death and may increase their migration through secretion of CXCL12. Finally, the authors proposed that targeting CAFs in MF may improve the efficiency of anti-cancer therapy [171].

### 1.22. Cytokines’ Influence on the Tumor Microenvironment’s Composition in MF/SS

Changes in the composition of the tumor microenvironment are driven by the release of cytokines from neoplastic cells. Although in early phases MF cells are few and interspersed between inflammatory milieu, they can acquire the ability to shift the immune system response from an anti-tumor (Th1) to a tumorigenic (Th2) one [172]. In brief, the shift from a Th1 to a Th2 inflammatory response will lead to an increase in immunosuppressive cytokine release (IL-2, IL-4, IL-7, IL-13, and IL-15) by neoplastic elements and tumor-associated cells, sustaining tumor growth and spread. As a consequence, there will be an accumulation of immature and immune-suppressive DCs and a depletion of mature (anti-tumor) ones as well as a recruitment of immunosuppressive cells (Treg cells and MDSCs) from blood vessels. Such a cascade of events will eventually empower tolerance and immune suppression by neoplastic cells, providing advantages to tumor growth and spread that are also driven by an increase in angiogenetic growth factor [87,172]. The overexpression of Th2 cytokines will also determine an overexpression of STAT3 and STAT5 pathways, which may be cytokine-dependent in early stages [173]. Changes in cytokine secretion in early and advanced MF/SS phases may pave the way to new target drugs able to restore an anti-tumor response and reverse the accumulation of immune-suppressive cells within the tumor microenvironment.

## 2. Materials and Methods

All of the literature concerning mycosis fungoides and microenvironment cells between 1950 and 2021 was examined. In particular, the keywords “mycosis fungoides” and “dendritic cells”, “mycosis fungoides” and “keratinocytes”, “mycosis fungoides” and “fibroblast”, “mycosis fungoides” and “endothelial cells”, “mycosis fungoides” and “tumor infiltrating lymphocytes”, “mycosis fungoides” and “eosinophils”, “mycosis fungoides” and “NK cells”, “mycosis fungoides” and “macrophages”, “mycosis fungoides” and “regulatory T and B cells”, and “mycosis fungoides” and “tumor associated macrophages” were searched for on PubMed. Data collected on the study were examined and elaborated in a narrative way.

## 3. Conclusions

The role of the microenvironment in MF progression is fascinating. Indeed, apart from genetic mutations [174,175,176], one alternative player involved in the progression from early to advanced lesions, the microenvironment may play a crucial role in MF and SS progression. Overall, microenvironment changes lead to immunosuppression given the presence of immature APCs or by the recruitment of immunosuppressive cells. Such a finding can pave the way to new therapeutic approaches focused on reversing the role of the microenvironment from an immunosuppressive to an anti-tumor one. From this point of view, some well-known drugs acting on the empowerment of the anti-tumor response are available: interferon-α (IFN- α) and bexarotene [177]. IFN-α directly enhances cell-mediated cytotoxicity and suppresses Th2 cytokine production by malignant T-cells. Geskin et al. observed a reduction in Treg cell and MDSC activity [26]. Bexarotene, by inducing malignant T-cell apoptosis and suppression of IL-4 production, may stimulate an anti-tumor response in responder patients [178]. A modest effect can also be obtained by retinoic acid receptor-specific (RAR-specific) administration (i.e., acitretin) by an increase in IL-12-dependent IFN- α production [177]. Another possible strategy to re-awaken the immune system is vaccination. Kim et al. [179] obtained systemic clinical responses in one-third of enrolled patients in a clinical trial based on the administration of intra-tumoral injection of a TLR9 agonist after a radiotherapeutic procedure. Most of the patients were highly treated non-responder patients. Currently, the tool-like receptor agonist seems to be a promising treatment focused on immune system empowerment. Imiquimod (a TLR7 agonist) works by inducing the release of a massive number of local cytokines, including type I IFNs, against MF. Anecdotal evidence seems quite promising, with a response rate ranging from 50% to 100% [180]. Other TLR agonists, such as topical resiquimod (a TLR7/8 agonist), have produced higher response rates (9 PR and 2 CR in 12 patients). Other experiences with the use of vaccines in MF are few, despite the attractive mechanism of action of sensitizing the host immune system against MF and SS neoantigens. Maier et al. [181] observed quite promising results (an ORR of 50%) with a vaccine of tumor-antigen-specific dendritic cells (generated by pulsing the cells in an autologous tumor lysate). Similar results were obtained by the use of attenuated virus after IFN-α treatment [182]. Extracorporeal photopheresis (ECP) is another treatment option that predominantly acts through immunomodulation, and its efficacy has been emphasized by 19 trials with over 400 patients [183]. By shifting from a Th2 to a Th1 cytokine pattern release, ECP plays a role in the anti-tumor host response. However, ECP is highly effective in SS owing to its ability to induce the apoptosis of circulating neoplastic cells. Another promising therapeutic scenario is represented by immunotherapy, although limited data are available. A phase I study of nivolumab demonstrated a 15% ORR in 13 patients [184]. Clinical trials on pembrolizumab in advanced-stage, highly pre-treated CTCLs showed a 56% and 27% ORR in MF and SS patients, respectively [185]. Only a case report of the application of ipilimumab to treat melanoma in a patient with concurrent MF is present in the literature [186]. There is an urgent unmet clinical need to identify the best candidates for immune therapy and also try to increase the response rates. Chimeric Antigen Receptor T-cells (CAR-T cells) may be another possible option. Indeed, CAR can be engineered to target specific antigens and to be inserted into T-cells to eliminate cells expressing those antigens. The principal problem in a T-cell malignancy is related to distinguishing between normal and tumor CAR. Two major problems are related to CAR-T cell use: mutual killing of CAR-T cells (fratricide) and T-cell aplasia induced by the destruction of normal T-cells. Another possible risk is contamination with circulating tumor T-cells when autologous T-cells are harvested to develop CAR-T cells [187]. The widely available evidence that TAMs are involved in CTCL development and associated with a poor prognosis suggests that macrophages can be a potential therapeutic target. Pharmacological drugs such as thalidomide, lenalidomide, pentoxifylline, and genistein are able to inhibit macrophage infiltration and reduce the tumor’s size, while other drugs can work against macrophage-induced angiogenesis, such as anti-VEGF-A and avastin/bevacizumab [64]. The therapeutic effects of bexarotene, a third-generation X receptor retinoid, are partially attributable to suppressive effects on the production of CCL22 by M2 TAMs [188]. Evidence of strong expression of CD30 on TAMs in MF and SS patients suggests that depletion of macrophages is one of the possible targets of the anti-CD30 monoclonal antibody Brentuximab vedotin [189]. TAMs also express PD-L1 and this expression negatively correlates with their phagocytic activity, so monoclonal antibodies that block the PD1/PDL1 axis were found to reduce tumor growth in macrophage-dependent fashion using in vitro and in vivo colon cancer models and improve macrophage-mediated T-cell activation in in vivo hepatocellular cancer studies [64,190,191]. Converting M2 macrophages into M1 macrophages could be a good goal for therapy and the use of activators of toll-like receptors has been proposed as a therapeutic strategy [64,192]. Moreover, paclitaxel, a plant-derived diterpenoid, can stimulate M1 macrophages, enhancing tumor cell cytotoxicity [64]. Due to MF’s indolent course and the frequency of relapses in MF and SS, treatments focused on the host immune system’s role are warranted. A recent finding showed that genetic aberrations and microenvironment cells may promote a transcriptional response fostering rapid malignant expansion, potentially influencing the response to scheduled treatments [193]. Knowledge of the role of the microenvironment and its interaction with neoplastic cells is an unmet need in the light of developing new treatment approaches.
